# *catena*-Poly[*N*,*N*,*N*′,*N*′-tetra­methyl­ethylendi­ammonium [[tetra­bromido­antimonate(III)]-μ-bromido] hemihydrate]

**DOI:** 10.1107/S1600536813028894

**Published:** 2013-11-06

**Authors:** Houda Kharrat, Slaheddine Kamoun, François Michaud

**Affiliations:** a Laboratoire de Génie des Matériaux et Environnement, Ecole Nationale d’Ingénieurs de Sfax, Université de Sfax, BP 1173, Sfax, Tunisia; b Service commun d’analyse par diffraction des rayons X, Université de Brest, 6 avenue Victor Le Gorgeu, CS 93837, F-29238 Brest Cedex 3, France

## Abstract

The asymmetric unit of the title compound {(C_6_H_18_N_2_)_2_[Sb_2_Br_10_]·H_2_O}_*n*_, consists of two tetra­methyl­ethylendi­ammonium cations that are located on centres of inversion, as well as one tetra­methyl­ethylendi­ammonium cation, one water mol­ecule, one distorted octahedral [SbBr_6_]^3−^anion and one bisphenoidal [SbBr_4_]^−^ anion in general positions. The [SbBr_6_]^3−^ and [SbBr_4_]^−^ anions are linked together by two long Sb—Br bonds of 3.2709 (8) and 3.5447 (7) Å into {[Sb_2_Br_10_]^4−^}_*n*_ chains along [001]. One of the three tetra­methyl­ethylendi­ammonium cations is disordered and was refined using a split model (occupancy ratio 0.58:0.42). The cations and the water mol­ecule are connected to the {[Sb_2_Br_10_]^4−^}_*n*_ polymeric anions by weak N—H ⋯Br and O(water)—H ⋯Br hydrogen bonding.

## Related literature

For crystal structures of related organic anti­monate(III) halogenides, see: Bujak & Angel (2005 ▶); Chaabouni *et al.* (1997 ▶, 1998 ▶). For a similar structure, see: Owczarek *et al.* (2012 ▶). The bond-valence sum was calculated using the parameters given by Brown & Altermatt (1985 ▶).
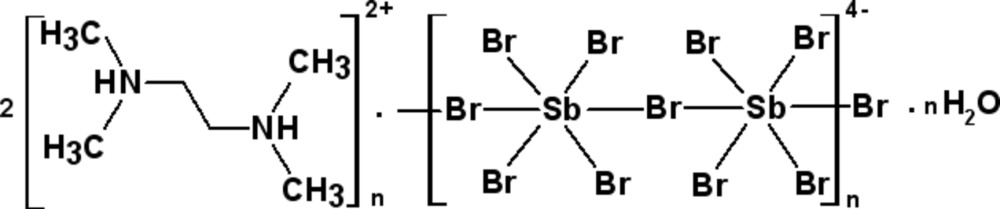


## Experimental

### 

#### Crystal data

(C_6_H_18_N_2_)[Sb_2_Br_10_]·H_2_O
*M* *_r_* = 1297.06
Orthorhombic, 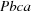

*a* = 18.0860 (4) Å
*b* = 19.1755 (4) Å
*c* = 19.4619 (4) Å
*V* = 6749.5 (2) Å^3^
*Z* = 8
Mo *K*α radiation
μ = 13.45 mm^−1^
*T* = 298 K
0.43 × 0.30 × 0.19 mm

#### Data collection

Oxford Diffraction Xcalibur Sapphire2 diffractometer
Absorption correction: multi-scan (*CrysAlis RED*; Oxford Diffraction, 2009 ▶) *T*_min_ = 0.011, *T*_max_ = 0.078
65748 measured reflections
10770 independent reflections
5721 reflections with *I* > 2σ(*I*)
*R*_int_ = 0.096

#### Refinement

*R*[*F* ^2^ > 2σ(*F* ^2^)] = 0.047
*wR*(*F* ^2^) = 0.089
*S* = 1.01
10770 reflections
290 parameters
15 restraints
H-atom parameters constrained
Δρ_max_ = 1.92 e Å^−3^
Δρ_min_ = −1.98 e Å^−3^

### 

Data collection: *CrysAlis CCD* (Oxford Diffraction, 2009 ▶); cell refinement: *CrysAlis RED* (Oxford Diffraction, 2009 ▶); data reduction: *CrysAlis RED*; program(s) used to solve structure: *SIR92* (Altomare *et al.*, 1993 ▶); program(s) used to refine structure: *SHELXL97* (Sheldrick, 2008 ▶); molecular graphics: *ORTEP-3 for Windows* (Farrugia, 2012 ▶) and *Mercury* (Macrae *et al.*, 2008 ▶); software used to prepare material for publication: *WinGX* (Farrugia, 2012 ▶) and *publCIF* (Westrip, 2010 ▶).

## Supplementary Material

Crystal structure: contains datablock(s) I. DOI: 10.1107/S1600536813028894/nc2318sup1.cif

Structure factors: contains datablock(s) I. DOI: 10.1107/S1600536813028894/nc2318Isup2.hkl

Additional supplementary materials:  crystallographic information; 3D view; checkCIF report

## Figures and Tables

**Table 1:** Hydrogen-bond geometry (Å, °)

*D*—H⋯*A*	*D*—H	H⋯*A*	*D*⋯*A*	*D*—H⋯*A*
N1—H1⋯Br1	0.91	2.59	3.362 (4)	143
N2—H2⋯Br1	0.91	2.56	3.353 (5)	146
N3—H3⋯Br7^i^	0.91	2.5	3.352 (4)	157
N4—H4⋯Br3	0.91	2.52	3.318 (4)	147
O*W*—H1*W*⋯Br4^ii^	0.83	3.03	3.759 (7)	148
O*W*—H2*W*⋯Br3	0.83	2.67	3.449 (7)	157

Symmetry codes: (i) 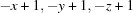; (ii) 
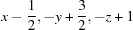
.

**Fig. 1.**
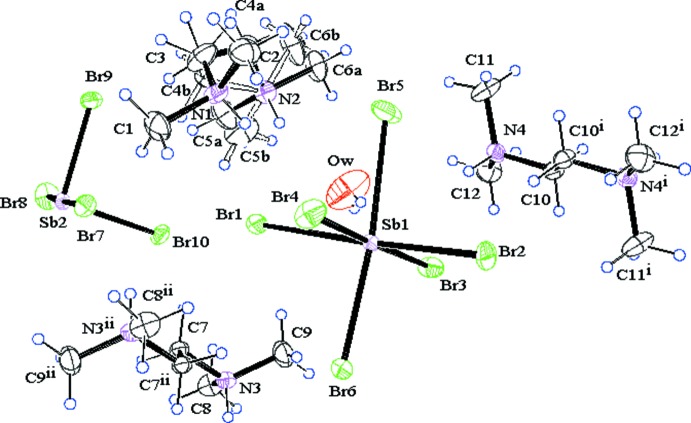
View of the asymmetric unit of the title compound with labelling and displacement ellipsoids drawn at the 30% probability level. Symmetry codes: (i) x + 1, -y + 2, -z + 1; (ii) -x + 1, -y + 1, -z + 1.

**Fig. 2.**
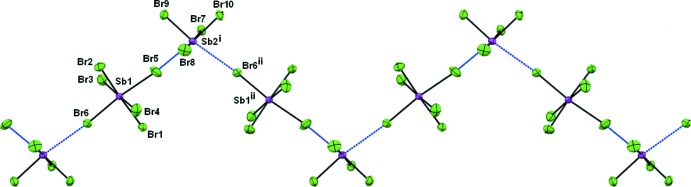
View of the anionic substructure of the title compound with labelling and displacement ellipsoids drawn at the 30% probability level. Symmetry codes: (i) -x + 1, y + 0.5, -z + 1.5; (ii) -x + 1, -y + 1, -z + 1.

## supplementary crystallographic information

### 1. Comment

The structure determination is part of a larger project related to the synthesis, structure and phase transitions in the group of new ferroic crystals of halogenoantimonates (III) with organic cations of various sizes and symmetries (Bujak & Angel, 2005; Chaabouni *et al.*, 1997; Chaabouni *et al.*, 1998). In these compounds the Sb atom shows a tendency toward distorded octahedral coordination with some rather long Sb—X bonds, which is attributed to the aspherical distribution of the lone pair electron (LP) at the Sb(III) cation.

The asymetric unit of the title compound consists of both, [SbBr_6_]^3-^ and [SbBr_4_]^-^ anions, a water molecule and three tetramethylethylene diammonium cations of which two are located on centers of inversion (Fig. 1). One of these cations is disordered and was refined using a split model. The anionic substructure is composed of distordered [Sb(1)Br_6_]^3-^ octahedra that share two *trans* corners with two others [Sb(2)Br_4_]^-^ anions that shows a saw-horse coordination. These anions are linked into zig zag {[Sb_2_Br_10_]^4-^}*_n_* pseudo chains that elongate along the [001] direction (Fig. 2). Two types of Sb—Br distances are present within these chains: eight short Sb—Br (terminal) distances [2.5405 (7) - 2.9906 (7) Å] and two long Sb—Br (bridging) distances [Sb(2)···Br(5) = 3.2709 (8) Å and Sb(2)···Br(6) = 3.5447 (7) Å], all of them are shorter than the sum of the Van der Waals radii (4.1 Å). A similar structural behavior was already reported by Owczarek (Owczarek *et al.*, 2012). By taking into account the sixth-fold coordination of antimony atom, we have proceeded to calculate the bond-valence sum (BVS) of this metal using the parameters given by Brown (Brown & Altermatt, 1985). The BVS calculation of the Sb(1) and Sb(2) ions confirm the presumed oxidation state of Sb (III). The difference between the longest and the shortest Sb—Br distances in the Sb(1)Br_6_ and Sb(2)Br_6_ units amount to 0.3353 (7) Å and 1.0042 (7) Å. Differences were also found in the Br—Sb—Br angles involving Br atoms that are mutually *cis* configurated. The differences are 13.79 (2)° for Sb(1)Br6 and 23.50 (2)° for Sb(2)Br6. Taking into account the differences described above the lone pair electron at the Sb(III) cations may be considered as stereochemical active. The [C_6_H_18_N_2_]^2+^ cations are located between the inorganic chains with their ammonium group facing the oppositely charged {[Sb_2_Br_10_]^4-^}*_n_* polyanions. In the crystal structure the cations, anions and water molecules are linked by weak intermolecular N—H···Br and O(W)—H···Br hydrogen bonding (Table 1).

### 2. Experimental

Crystals of the title compound were obtained by dissolving a stoichiometric mixture of antimony (III) oxide Sb_2_O_3_ (5 g, 17 mmol) and N, N, N', N' - tetramethylethylendiamine (C_6_H_16_N_2_) (5 ml, 34 mmol) in 100 ml of a solution of HBr (24%) . The resulting aqueous solution was then kept at room temperature. After several weeks prismatic shaped single crystals of the title compound were obtained by slow evaporation of the solvent at room temperature. They were washed with diethyl ether and dried for 4 h over CaCl_2_.

### 3. Refinement

All C-H and N-H H atoms were positioned with idealized geometry and refined with *U*_iso_(H) = 1.2 *U*_eq_(*C,N*) (1.5 for methyl H atoms) using a riding model with C-H = 0.96 Å for methyl, C-H = 0.97 Å for methylene and N—H = 0.91 Å for ammonium H atoms. The H atoms of the water molecules were located in difference map, their bond lengths were set to ideal values and finally they were refined using a riding model with *U*_iso_(H) = 1.5 *U*_eq_(*O*). The tetramethylethylendiammonium cation in a general position is disordered and was refined using a split model with occupancy ratio 58:42 using restraints. The O atom of the water molecule shows slightly enlarged anisotropic displacement parameters indicating for some disordering that cannot be resolved successfully.

### Crystal data

**Table:**

(C_6_H_18_N_2_)[Sb_2_Br_10_]·H_2_O	*Z* = 8
*M**_r_* = 1297.06	*F*(000) = 4784
Orthorhombic, *P**b**c**a*	*D*_x_ = 2.553 Mg m^−^^3^
Hall symbol: -P 2ac 2ab	Mo *K*α radiation, λ = 0.71073 Å
*a* = 18.0860 (4) Å	µ = 13.45 mm^−^^1^
*b* = 19.1755 (4) Å	*T* = 298 K
*c* = 19.4619 (4) Å	Prismatic, axis [1 0 0], yellow
*V* = 6749.5 (2) Å^3^	0.43 × 0.30 × 0.19 mm

### Data collection

**Table:**

Oxford Diffraction Xcalibur Sapphire2 diffractometer	10770 independent reflections
Radiation source: sealed X-ray tube	5721 reflections with *I* > 2σ(*I*)
Graphite monochromator	*R*_int_ = 0.096
Detector resolution: 8.3622 pixels mm^-1^	θ_max_ = 31.6°, θ_min_ = 3.1°
ω scans	*h* = −25→26
Absorption correction: multi-scan (*CrysAlis RED*; Oxford Diffraction, 2009)	*k* = −28→26
*T*_min_ = 0.011, *T*_max_ = 0.078	*l* = −26→28
65748 measured reflections	

### Refinement

**Table:**

Refinement on *F*^2^	Primary atom site location: structure-invariant direct methods
Least-squares matrix: full	Secondary atom site location: difference Fourier map
*R*[*F*^2^ > 2σ(*F*^2^)] = 0.047	Hydrogen site location: inferred from neighbouring sites
*wR*(*F*^2^) = 0.089	H-atom parameters constrained
*S* = 1.01	*w* = 1/[σ^2^(*F*_o_^2^) + (0.033*P*)^2^ + 2.8328*P*] where *P* = (*F*_o_^2^ + 2*F*_c_^2^)/3
10770 reflections	(Δ/σ)_max_ = 0.001
290 parameters	Δρ_max_ = 1.92 e Å^−^^3^
15 restraints	Δρ_min_ = −1.98 e Å^−^^3^

### Special details

**Table:**

Experimental. Empirical absorption correction using spherical harmonics, implemented in SCALE3 ABSPACK scaling algorithm. CrysAlis RED (Oxford Diffraction, 2009)
Geometry. All s.u.'s (except the s.u. in the dihedral angle between two l.s. planes) are estimated using the full covariance matrix. The cell s.u.'s are taken into account individually in the estimation of s.u.'s in distances, angles and torsion angles; correlations between s.u.'s in cell parameters are only used when they are defined by crystal symmetry. An approximate (isotropic) treatment of cell s.u.'s is used for estimating s.u.'s involving l.s. planes.
Refinement. Refinement of *F*^2^ against ALL reflections. The weighted *R*-factor *wR* and goodness of fit *S* are based on *F*^2^, conventional *R*-factors *R* are based on *F*, with *F* set to zero for negative *F*^2^. The threshold expression of *F*^2^ > σ(*F*^2^) is used only for calculating *R*-factors(gt) *etc*. and is not relevant to the choice of reflections for refinement. *R*-factors based on *F*^2^ are statistically about twice as large as those based on *F*, and *R*- factors based on ALL data will be even larger.

### Fractional atomic coordinates and isotropic or equivalent isotropic displacement parameters (Å^2^)

**Table:**

	*x*	*y*	*z*	*U*_iso_*/*U*_eq_	Occ. (<1)
Sb1	0.626802 (19)	0.755771 (18)	0.520272 (17)	0.02825 (9)	
Sb2	0.351898 (18)	0.442769 (18)	0.734954 (18)	0.02673 (9)	
Br1	0.54458 (4)	0.65129 (3)	0.59658 (3)	0.04546 (16)	
Br2	0.70124 (4)	0.85628 (3)	0.45458 (4)	0.05252 (18)	
Br3	0.48610 (3)	0.81097 (4)	0.46063 (3)	0.04992 (18)	
Br4	0.75101 (4)	0.70887 (4)	0.57645 (3)	0.0591 (2)	
Br5	0.59238 (4)	0.83959 (4)	0.64163 (4)	0.0646 (2)	
Br6	0.64208 (4)	0.66424 (3)	0.41346 (3)	0.04907 (17)	
Br7	0.50362 (3)	0.48013 (3)	0.70652 (3)	0.03898 (15)	
Br8	0.21218 (3)	0.41314 (4)	0.76132 (4)	0.0591 (2)	
Br9	0.35219 (3)	0.53266 (3)	0.83085 (3)	0.04420 (16)	
Br10	0.31213 (3)	0.53122 (3)	0.64461 (3)	0.04336 (16)	
N1	0.6426 (2)	0.6779 (3)	0.7410 (2)	0.0377 (12)	
H1	0.6259	0.6911	0.6989	0.045*	
N2	0.4676 (3)	0.7303 (3)	0.7326 (3)	0.0423 (12)	
H2	0.4908	0.7287	0.6911	0.051*	
C1	0.6693 (4)	0.6051 (3)	0.7355 (4)	0.061 (2)	
H1A	0.6291	0.5753	0.7222	0.091*	
H1B	0.6883	0.5902	0.7791	0.091*	
H1C	0.7077	0.6026	0.7015	0.091*	
C2	0.7034 (4)	0.7249 (4)	0.7603 (4)	0.065 (2)	
H2A	0.6851	0.7718	0.7633	0.098*	
H2B	0.7416	0.7225	0.7262	0.098*	
H2C	0.723	0.711	0.804	0.098*	
C3	0.5793 (4)	0.6819 (4)	0.7901 (3)	0.066 (2)	
H3A	0.5544	0.6372	0.7883	0.08*	0.58
H3B	0.6004	0.6859	0.8357	0.08*	0.58
H3C	0.5908	0.6497	0.827	0.08*	0.42
H3D	0.5812	0.7282	0.8099	0.08*	0.42
C4A	0.5236 (6)	0.7339 (6)	0.7848 (5)	0.047 (3)	0.58
H4A	0.5486	0.7783	0.7793	0.056*	0.58
H4B	0.4984	0.7356	0.8288	0.056*	0.58
C5A	0.4178 (12)	0.6752 (8)	0.7351 (13)	0.071 (6)	0.58
H5A	0.3847	0.6781	0.6966	0.107*	0.58
H5B	0.3899	0.6776	0.777	0.107*	0.58
H5C	0.4443	0.6319	0.7333	0.107*	0.58
C6A	0.4223 (7)	0.8015 (6)	0.7363 (7)	0.058 (4)	0.58
H6A	0.4559	0.8402	0.7345	0.087*	0.58
H6B	0.3947	0.8032	0.7784	0.087*	0.58
H6C	0.3888	0.8041	0.6981	0.087*	0.58
C4B	0.5103 (7)	0.6709 (6)	0.7744 (6)	0.031 (3)	0.42
H4C	0.4835	0.6626	0.8167	0.037*	0.42
H4D	0.5079	0.6282	0.7476	0.037*	0.42
C5B	0.3998 (18)	0.6842 (15)	0.7092 (19)	0.080 (10)	0.42
H5E	0.417	0.6468	0.6807	0.12*	0.42
H5F	0.3656	0.7125	0.6838	0.12*	0.42
H5G	0.3756	0.6654	0.749	0.12*	0.42
C6B	0.4477 (2)	0.7858 (2)	0.7706 (2)	0.092 (9)	0.42
H6E	0.4911	0.8112	0.784	0.138*	0.42
H6F	0.4219	0.7702	0.8109	0.138*	0.42
H6G	0.4159	0.8154	0.7441	0.138*	0.42
N3	0.4164 (2)	0.5295 (2)	0.4486 (2)	0.0347 (11)	
H3	0.4377	0.5133	0.4094	0.042*	
C7	0.4592 (3)	0.4993 (3)	0.5075 (3)	0.0318 (13)	
H7A	0.4436	0.4515	0.5154	0.038*	
H7B	0.4492	0.5259	0.5489	0.038*	
C8	0.3390 (3)	0.5050 (4)	0.4484 (3)	0.0513 (18)	
H8A	0.3381	0.4551	0.4524	0.077*	
H8B	0.3156	0.5187	0.4062	0.077*	
H8C	0.313	0.5253	0.4865	0.077*	
C9	0.4198 (4)	0.6065 (3)	0.4455 (4)	0.0596 (19)	
H9A	0.389	0.6229	0.4087	0.089*	
H9B	0.4699	0.6209	0.4376	0.089*	
H9C	0.4027	0.6256	0.4882	0.089*	
N4	0.4240 (2)	0.9494 (2)	0.5451 (2)	0.0348 (11)	
H4	0.4463	0.9071	0.5405	0.042*	
C10	0.4578 (3)	0.9977 (3)	0.4958 (3)	0.0373 (14)	
H10A	0.4366	1.0437	0.502	0.045*	
H10B	0.4464	0.9824	0.4495	0.045*	
C11	0.4316 (4)	0.9720 (4)	0.6179 (3)	0.064 (2)	
H11A	0.4058	0.9399	0.6472	0.095*	
H11B	0.411	1.0178	0.6232	0.095*	
H11C	0.483	0.9728	0.6302	0.095*	
C12	0.3444 (3)	0.9408 (4)	0.5294 (4)	0.061 (2)	
H12A	0.3231	0.9076	0.5605	0.091*	
H12B	0.3389	0.9243	0.4831	0.091*	
H12C	0.3198	0.9848	0.5343	0.091*	
OW	0.3596 (4)	0.7703 (5)	0.5870 (4)	0.159 (3)	
H1W	0.3219	0.7677	0.563	0.238*	
H2W	0.3989	0.7757	0.566	0.238*	

### Atomic displacement parameters (Å^2^)

**Table:**

	*U*^11^	*U*^22^	*U*^33^	*U*^12^	*U*^13^	*U*^23^
Sb1	0.0396 (2)	0.02096 (19)	0.02414 (17)	−0.00123 (16)	0.00398 (16)	−0.00193 (15)
Sb2	0.02741 (18)	0.02436 (19)	0.02841 (18)	0.00197 (15)	−0.00144 (15)	0.00237 (16)
Br1	0.0683 (4)	0.0336 (3)	0.0345 (3)	−0.0137 (3)	0.0033 (3)	−0.0023 (3)
Br2	0.0636 (4)	0.0405 (4)	0.0535 (4)	−0.0205 (3)	0.0045 (3)	0.0098 (3)
Br3	0.0512 (4)	0.0541 (4)	0.0444 (4)	0.0098 (3)	−0.0033 (3)	−0.0109 (3)
Br4	0.0503 (4)	0.0776 (5)	0.0493 (4)	0.0205 (4)	−0.0013 (3)	0.0034 (4)
Br5	0.0788 (5)	0.0500 (4)	0.0650 (5)	−0.0101 (4)	0.0015 (4)	−0.0271 (4)
Br6	0.0785 (5)	0.0318 (3)	0.0369 (3)	−0.0005 (3)	0.0089 (3)	−0.0031 (3)
Br7	0.0333 (3)	0.0463 (4)	0.0373 (3)	−0.0007 (3)	0.0038 (2)	0.0046 (3)
Br8	0.0324 (3)	0.0653 (5)	0.0795 (5)	−0.0106 (3)	0.0040 (3)	0.0040 (4)
Br9	0.0542 (4)	0.0467 (4)	0.0318 (3)	0.0074 (3)	−0.0061 (3)	−0.0070 (3)
Br10	0.0519 (4)	0.0484 (4)	0.0298 (3)	0.0162 (3)	−0.0033 (3)	0.0071 (3)
N1	0.036 (3)	0.050 (3)	0.027 (2)	0.008 (2)	−0.004 (2)	−0.001 (2)
N2	0.033 (3)	0.048 (3)	0.046 (3)	0.008 (2)	0.003 (2)	−0.002 (3)
C1	0.060 (5)	0.045 (4)	0.078 (5)	0.021 (3)	−0.009 (4)	−0.005 (4)
C2	0.067 (5)	0.068 (5)	0.060 (5)	−0.019 (4)	0.001 (4)	−0.008 (4)
C3	0.061 (5)	0.100 (7)	0.038 (4)	0.028 (4)	0.011 (3)	0.012 (4)
C4A	0.048 (7)	0.052 (8)	0.039 (6)	0.019 (6)	0.002 (5)	−0.007 (6)
C5A	0.054 (13)	0.050 (9)	0.11 (2)	−0.011 (8)	0.003 (10)	−0.008 (11)
C6A	0.042 (7)	0.041 (7)	0.092 (10)	0.021 (6)	−0.007 (7)	0.008 (7)
C4B	0.050 (9)	0.015 (7)	0.028 (7)	0.004 (6)	0.002 (6)	−0.008 (6)
C5B	0.041 (15)	0.12 (2)	0.07 (2)	−0.020 (13)	−0.018 (11)	0.002 (16)
C6B	0.056 (14)	0.034 (11)	0.19 (3)	0.023 (10)	0.029 (15)	0.003 (15)
N3	0.036 (3)	0.039 (3)	0.029 (3)	−0.001 (2)	0.003 (2)	−0.006 (2)
C7	0.036 (3)	0.034 (3)	0.025 (3)	−0.004 (3)	0.004 (2)	−0.003 (3)
C8	0.031 (3)	0.073 (5)	0.050 (4)	0.002 (3)	−0.003 (3)	−0.002 (4)
C9	0.073 (5)	0.037 (4)	0.068 (5)	0.009 (3)	−0.012 (4)	0.005 (4)
N4	0.038 (3)	0.028 (3)	0.039 (3)	0.008 (2)	0.006 (2)	−0.001 (2)
C10	0.038 (3)	0.045 (4)	0.029 (3)	−0.002 (3)	−0.003 (3)	0.000 (3)
C11	0.062 (4)	0.100 (6)	0.028 (3)	−0.003 (4)	0.001 (3)	0.006 (4)
C12	0.050 (4)	0.065 (5)	0.067 (5)	−0.010 (4)	0.002 (3)	−0.006 (4)
OW	0.128 (6)	0.243 (10)	0.105 (5)	−0.005 (6)	−0.008 (5)	0.028 (6)

### Geometric parameters (Å, º)

**Table:**

Sb1—Br1	2.9035 (7)	C6A—H6A	0.96
Sb1—Br2	2.6762 (7)	C6A—H6B	0.96
Sb1—Br3	2.9906 (7)	C6A—H6C	0.96
Sb1—Br4	2.6553 (7)	C4B—H4C	0.97
Sb1—Br5	2.9240 (8)	C4B—H4D	0.97
Sb1—Br6	2.7347 (7)	C5B—H5E	0.96
Br5—Sb2^i^	3.2709 (8)	C5B—H5F	0.96
Sb2—Br6^ii^	3.5447 (7)	C5B—H5G	0.96
Sb2—Br7	2.8895 (6)	C6B—H6E	0.96
Sb2—Br8	2.6403 (7)	C6B—H6F	0.96
Sb2—Br9	2.5405 (7)	C6B—H6G	0.96
Sb2—Br10	2.5466 (7)	N3—C8	1.477 (7)
N1—C2	1.471 (7)	N3—C9	1.479 (7)
N1—C1	1.480 (7)	N3—C7	1.500 (6)
N1—C3	1.493 (8)	N3—H3	0.91
N1—H1	0.91	C7—C7^ii^	1.503 (10)
N2—C6B	1.345 (7)	C7—H7A	0.97
N2—C5A	1.389 (19)	C7—H7B	0.97
N2—C4A	1.437 (10)	C8—H8A	0.96
N2—C5B	1.58 (2)	C8—H8B	0.96
N2—C6A	1.596 (11)	C8—H8C	0.96
N2—C4B	1.598 (13)	C9—H9A	0.96
N2—H2	0.91	C9—H9B	0.96
C1—H1A	0.96	C9—H9C	0.96
C1—H1B	0.96	N4—C10	1.467 (7)
C1—H1C	0.96	N4—C12	1.480 (7)
C2—H2A	0.96	N4—C11	1.487 (7)
C2—H2B	0.96	N4—H4	0.91
C2—H2C	0.96	C10—C10^iii^	1.537 (10)
C3—C4B	1.302 (13)	C10—H10A	0.97
C3—C4A	1.422 (11)	C10—H10B	0.97
C3—H3A	0.97	C11—H11A	0.96
C3—H3B	0.97	C11—H11B	0.96
C3—H3C	0.97	C11—H11C	0.96
C3—H3D	0.97	C12—H12A	0.96
C4A—H4A	0.97	C12—H12B	0.96
C4A—H4B	0.97	C12—H12C	0.96
C5A—H5A	0.96	OW—H1W	0.828
C5A—H5B	0.96	OW—H2W	0.8264
C5A—H5C	0.96		
			
Br1—Sb1—Br2	177.35 (2)	H3A—C3—H3D	147.2
Br1—Sb1—Br3	90.40 (2)	H3B—C3—H3D	63.1
Br1—Sb1—Br4	89.37 (2)	H3C—C3—H3D	106.3
Br1—Sb1—Br5	81.78 (2)	C3—C4A—N2	121.0 (9)
Br1—Sb1—Br6	89.87 (2)	C3—C4A—H4A	107.1
Br2—Sb1—Br3	89.30 (2)	N2—C4A—H4A	107.1
Br2—Sb1—Br4	90.86 (3)	C3—C4A—H4B	107.1
Br2—Sb1—Br5	95.57 (2)	N2—C4A—H4B	107.1
Br2—Sb1—Br6	92.77 (2)	H4A—C4A—H4B	106.8
Br3—Sb1—Br4	178.37 (2)	N2—C5A—H5A	109.5
Br3—Sb1—Br5	86.43 (2)	N2—C5A—H5B	109.5
Br3—Sb1—Br6	91.03 (2)	H5A—C5A—H5B	109.5
Br4—Sb1—Br5	91.93 (2)	N2—C5A—H5C	109.5
Br4—Sb1—Br6	90.58 (2)	H5A—C5A—H5C	109.5
Br5—Sb1—Br6	171.25 (2)	H5B—C5A—H5C	109.5
Br5^iv^—Sb2—Br6^ii^	103.81 (2)	N2—C6A—H6A	109.5
Br5^iv^—Sb2—Br7	89.89 (2)	N2—C6A—H6B	109.5
Br5^iv^—Sb2—Br8	91.26 (2)	H6A—C6A—H6B	109.5
Br5^iv^—Sb2—Br9	82.55 (2)	N2—C6A—H6C	109.5
Br5^iv^—Sb2—Br10	175.44 (2)	H6A—C6A—H6C	109.5
Br6^ii^—Sb2—Br7	87.61 (2)	H6B—C6A—H6C	109.5
Br6^ii^—Sb2—Br8	93.62 (2)	C3—C4B—N2	117.8 (10)
Br6^ii^—Sb2—Br9	172.43 (2)	C3—C4B—H4C	107.9
Br6^ii^—Sb2—Br10	80.31 (2)	N2—C4B—H4C	107.9
Br7—Sb2—Br8	178.07 (3)	C3—C4B—H4D	107.9
Br7—Sb2—Br9	88.31 (2)	N2—C4B—H4D	107.9
Br7—Sb2—Br10	88.33 (2)	H4C—C4B—H4D	107.2
Br8—Sb2—Br9	90.30 (2)	N2—C5B—H5E	109.5
Br8—Sb2—Br10	90.41 (2)	N2—C5B—H5F	109.5
Br9—Sb2—Br10	93.20 (2)	H5E—C5B—H5F	109.5
Sb2^i^—Br5—Sb1	149.32 (3)	N2—C5B—H5G	109.5
Sb2^ii^—Br6—Sb1	173.75 (3)	H5E—C5B—H5G	109.5
C2—N1—C1	110.7 (5)	H5F—C5B—H5G	109.5
C2—N1—C3	112.2 (5)	N2—C6B—H6E	109.5
C1—N1—C3	110.2 (5)	N2—C6B—H6F	109.5
C2—N1—H1	107.9	H6E—C6B—H6F	109.5
C1—N1—H1	107.9	N2—C6B—H6G	109.5
C3—N1—H1	107.9	H6E—C6B—H6G	109.5
C6B—N2—C5A	114.1 (12)	H6F—C6B—H6G	109.5
C6B—N2—C4A	76.2 (5)	C8—N3—C9	110.9 (5)
C5A—N2—C4A	118.1 (10)	C8—N3—C7	111.6 (4)
C6B—N2—C5B	113.2 (15)	C9—N3—C7	113.3 (4)
C5A—N2—C5B	23.1 (17)	C8—N3—H3	106.9
C4A—N2—C5B	141.2 (13)	C9—N3—H3	106.9
C6B—N2—C6A	32.9 (5)	C7—N3—H3	106.9
C5A—N2—C6A	108.4 (10)	N3—C7—C7^ii^	110.5 (5)
C4A—N2—C6A	106.8 (7)	N3—C7—H7A	109.6
C5B—N2—C6A	95.3 (13)	C7^ii^—C7—H7A	109.6
C6B—N2—C4B	114.4 (6)	N3—C7—H7B	109.6
C5A—N2—C4B	75.8 (9)	C7^ii^—C7—H7B	109.6
C4A—N2—C4B	48.3 (6)	H7A—C7—H7B	108.1
C5B—N2—C4B	97.1 (11)	N3—C8—H8A	109.5
C6A—N2—C4B	146.8 (8)	N3—C8—H8B	109.5
C6B—N2—H2	129.6	H8A—C8—H8B	109.5
C5A—N2—H2	107.7	N3—C8—H8C	109.5
C4A—N2—H2	107.7	H8A—C8—H8C	109.5
C5B—N2—H2	94.8	H8B—C8—H8C	109.5
C6A—N2—H2	107.7	N3—C9—H9A	109.5
C4B—N2—H2	101.8	N3—C9—H9B	109.5
N1—C1—H1A	109.5	H9A—C9—H9B	109.5
N1—C1—H1B	109.5	N3—C9—H9C	109.5
H1A—C1—H1B	109.5	H9A—C9—H9C	109.5
N1—C1—H1C	109.5	H9B—C9—H9C	109.5
H1A—C1—H1C	109.5	C10—N4—C12	109.9 (4)
H1B—C1—H1C	109.5	C10—N4—C11	113.6 (5)
N1—C2—H2A	109.5	C12—N4—C11	108.7 (5)
N1—C2—H2B	109.5	C10—N4—H4	108.2
H2A—C2—H2B	109.5	C12—N4—H4	108.2
N1—C2—H2C	109.5	C11—N4—H4	108.2
H2A—C2—H2C	109.5	N4—C10—C10^iii^	112.4 (6)
H2B—C2—H2C	109.5	N4—C10—H10A	109.1
C4B—C3—C4A	54.4 (7)	C10^iii^—C10—H10A	109.1
C4B—C3—N1	125.2 (8)	N4—C10—H10B	109.1
C4A—C3—N1	122.3 (7)	C10^iii^—C10—H10B	109.1
C4B—C3—H3A	53.3	H10A—C10—H10B	107.9
C4A—C3—H3A	106.8	N4—C11—H11A	109.5
N1—C3—H3A	106.8	N4—C11—H11B	109.5
C4B—C3—H3B	127.2	H11A—C11—H11B	109.5
C4A—C3—H3B	106.8	N4—C11—H11C	109.5
N1—C3—H3B	106.8	H11A—C11—H11C	109.5
H3A—C3—H3B	106.6	H11B—C11—H11C	109.5
C4B—C3—H3C	106	N4—C12—H12A	109.5
C4A—C3—H3C	130.7	N4—C12—H12B	109.5
N1—C3—H3C	106	H12A—C12—H12B	109.5
H3A—C3—H3C	64.1	N4—C12—H12C	109.5
H3B—C3—H3C	44.6	H12A—C12—H12C	109.5
C4B—C3—H3D	106	H12B—C12—H12C	109.5
C4A—C3—H3D	53.9	H1W—OW—H2W	115.9
N1—C3—H3D	106		

Symmetry codes: (i) −*x*+1, *y*+1/2, −*z*+3/2; (ii) −*x*+1, −*y*+1, −*z*+1; (iii) −*x*+1, −*y*+2, −*z*+1; (iv) −*x*+1, *y*−1/2, −*z*+3/2.

### Hydrogen-bond geometry (Å, º)

**Table:**

*D*—H···*A*	*D*—H	H···*A*	*D*···*A*	*D*—H···*A*
N1—H1···Br1	0.91	2.59	3.362 (4)	143
N2—H2···Br1	0.91	2.56	3.353 (5)	146
N3—H3···Br7^ii^	0.91	2.5	3.352 (4)	157
N4—H4···Br3	0.91	2.52	3.318 (4)	147
O*W*—H1*W*···Br4^v^	0.83	3.03	3.759 (7)	148
O*W*—H2*W*···Br3	0.83	2.67	3.449 (7)	157

Symmetry codes: (ii) −*x*+1, −*y*+1, −*z*+1; (v) *x*−1/2, −*y*+3/2, −*z*+1.
